# Postpartum women’s psychological experiences during the COVID-19 pandemic: a modified recurrent cross-sectional thematic analysis

**DOI:** 10.1186/s12884-021-04071-2

**Published:** 2021-09-17

**Authors:** Leanne Jackson, Leonardo De Pascalis, Joanne A. Harrold, Victoria Fallon, Sergio A. Silverio

**Affiliations:** 1 Department of Psychology, Institute of Population Health, Faculty of Health and Life Sciences, University of Liverpool, Eleanor Rathbone Building, Bedford Street South, Liverpool, Merseyside L69 7ZA UK; 2 Department of Women & Children’s Health, School of Life Course Sciences, Faculty of Life Sciences & Medicine, King’s College London, London, UK

**Keywords:** COVID-19, Postnatal care, Burnout, psychological, Psychosocial support systems, Mental health services, Psychological distress

## Abstract

**Background:**

COVID-19 has placed additional stressors on mothers during an already vulnerable lifecourse transition. Initial social distancing restrictions (Timepoint 1; T1) and initial changes to those social distancing restrictions (Timepoint 2; T2) have disrupted postpartum access to practical and emotional support. This qualitative study explores the postpartum psychological experiences of UK women during different phases of the COVID-19 pandemic and associated ‘lockdowns’.

**Methods:**

Semi-structured interviews were conducted with 12 women, approximately 30 days after initial social distancing guidelines were imposed in the UK (22 April 2020). A separate 12 women were interviewed approximately 30 days after the initial easing of social distancing restrictions (10 June 2020). Data were transcribed verbatim, uploaded into NVivo for management and analysis, which followed a recurrent cross-sectional approach to thematic analysis.

**Results:**

Two main themes were identified for T1: ‘*Motherhood is Much Like Lockdown*’ and ‘*A Self-Contained Family Unit*’. Each main T1 theme contained two sub-themes. Two main themes were also identified for T2: *‘Incongruously Held Views of COVID-19’* and *‘Mothering Amidst the Pandemic’.* Each main T2 theme contained three sub-themes. Comparisons between data gathered at each timepoint identified increased emotional distress over time. Current findings call for the improvement of postpartum care by improving accessibility to social support, and prioritising the re-opening of schools, and face-to-face healthcare appointments and visitation.

**Conclusion:**

Social distancing restrictions associated with COVID-19 have had a cumulative, negative effect on postpartum mental health. Recommendations such as: Allowing mothers to ‘bubble’ with a primary support provider even at their healthcare appointments; allowing one support partner to attend all necessary healthcare appointments; and providing tailored informational resources, may help to support postpartum emotional wellbeing during this, and similar health crises in the future.

**Supplementary Information:**

The online version contains supplementary material available at 10.1186/s12884-021-04071-2.

## Background

### COVID-19: a Global Health crisis

The global Coronavirus [SARS-CoV-2] pandemic or ‘COVID-19’ was first detected in Wuhan, China, in November 2019, as a respiratory disease with varying symptom severity [1, 2]. On 30 January 2020, COVID-19 was declared a public health emergency of international concern [3]. Mortality rates are relatively low for individuals under 65 years of age and for those without underlying health conditions, while mortality rates are higher for people who have significant clinical vulnerabilities [4, 5]. Women in the third trimester of pregnancy and those in the early postnatal period were initially thought to be more susceptible to contracting the virus [1, 6], and were advised to ‘shield’ (remain at home under all circumstances unless seeking urgent medical care, fleeing danger, or seeking medical attention for the birth of their baby [1, 2, 6]). Though this guidance was later revoked by the Royal College of Obstetricians and Gynaecologists (RCOG). However, women in their final trimester of pregnancy who contract COVID-19 have been found to experience more severe negative outcomes, compared with the general public [1, 7].

### COVID-19 and social distancing restrictions

Due to growing concerns regarding the spread and mortality rates associated with COVID-19, a nationwide ‘lockdown’ (stay-at-home order) was imposed by the UK Government on 23 March 2020 [6]. During this time the following recommendations were made for pregnant and postnatal women [8]: A minimum of six face-to-face antenatal consultations and a minimum of three postnatal contacts advised (remote where possible); a telephone call should be made prior to face-to-face appointments, to ensure mothers were not displaying COVID-19 symptoms; appointments should be consolidated where possible, to reduce potential exposure to COVID-19; non-hospital induction and labour should be considered for low-risk women; one birth partner only, permitted at hospital births, from active labour onwards; after giving birth, normal COVID-19 visitation restrictions are implemented and the mother’s selected birth partner would then be asked to leave; and for women who have experienced a late miscarriage (where a baby dies at between 14^+ 0^ – 23^+ 6^ weeks of gestation) or a stillbirth (where a baby dies at or after 24^+ 0^ weeks of gestation) one partner has been permitted throughout labour and until the mother is discharged from hospital.

### COVID-19 and postnatal vulnerabilities

The postnatal period marks a major transition in a woman’s lifecourse, which can alter many aspects of life, including: one’s identity to include motherhood [9], relationship satisfaction [10], and lifestyle [11]. Such a major transition may, in part, explain the heightened risk of experiencing mental distress during this period [12]. Transitioning into new motherhood amidst the COVID-19 pandemic poses unique stressors which may exacerbate an already vulnerable period in a woman’s life [13]. A recent qualitative study which examined perinatal experiences during COVID-19 via free-text responses, found that virtual consultations from healthcare professionals were viewed as impersonal and led to women feeling too embarrassed to discuss mental health concerns [14].

A large online survey sought to examine women’s psychosocial experiences during COVID-19 [15]. This research [15] found, despite prevalence of self-reported clinically diagnosed anxiety (18.4%) and depression (11.4%) reflecting pre-pandemic levels [12], a concerning proportion of mothers scored above clinically relevant cut-offs for anxiety (61%) and depression (43%). This suggests a worrisome proportion of mothers may be insufficiently supported for their level of need. There is little existing qualitative literature which has sought to explore women’s psychological experiences during the COVID-19 pandemic. Most available literature has focused exclusively on the initial phase of UK lockdown restrictions. Qualitative research can convey richer insight into which components of social distancing restrictions have been most impactful to maternal emotional wellbeing. The current study therefore aims to extend deeper understandings to recently published, quantitative works which have examined psychological experiences of motherhood during the COVID-19 pandemic [15] through in-depth qualitative analysis. More broadly, the current study aims to contribute towards the existing literature-base, by exploring the postpartum psychological experiences of UK women during the COVID-19 pandemic.

## Methods

### Ethical statement

Ethical approval was obtained from the University of Liverpool Central University Research Ethics Committee on 7 April 2020 (Project ID: IPHS/7630).

#### Design

A qualitative research design was utilised, with individual semi-structured interviews as the method of data collection. Interviews were conducted (LJ), via telephone or using video-calling (e.g., Zoom). Timepoint One (T1) interviews commenced on 22 April 2020, approximately thirty days after the introduction of initial social distancing restrictions (23 March 2020 [6];). Timepoint Two (T2) interviews commenced on the 10 June 2020, approximately thirty days after the initial easing of social distancing restrictions (11 May 2020 [6]). There were twelve women recruited for T1 (completed 20 May 2020); and a different sample of twelve women recruited for T2 (completed 16 July 2020). The interview schedule was created with collaborators who have experience in the field of maternal mental health [VF, SAS, JAH, LDP]. Interview questions were chronological, and therefore split by time period. For T1 interviews, this included discussions about: before COVID-19, the time around the interview, the future; and then general opinions about COVID-19. For T2 interviews, this included discussions on: the start of lockdown restrictions being implemented on 23 March 2020, the time around the interview, the future; and then general opinions about COVID-19. Please see supplementary documentation for T1 and T2 interview schedules.

#### Participants

To take part in the current study, participants needed to be 18 years of age or over, English speaking, and had to have given birth to a live infant within the past 3 months prior to the interview [15]. Participants had to have given birth in the UK as well as being currently resident in the UK at the time of the interview due to cross-country differences in lockdown restrictions [16]. All participants met necessary inclusion criteria.

The current qualitative study was nested within a larger, on-line, survey study exploring psychosocial experiences of new mothers during COVID-19 [15]. Participants who had been debriefed after taking part in the previously detailed survey studies were then automatically re-directed to a separate Qualtrics survey. This separate Qualtrics survey included a question which asked if the participant would be interested in taking part in an audio recorded interview study (if yes, they were then asked to provide their email address and/or telephone number so that the research team could contact them with more information).

Initial interest in taking part in an audio recorded interview study was expressed by 221 and 207 women at T1 and T2, respectively. Due to interest being oversubscribed, a random number generator was used to select potential participants to approach (LJ). Selected participants were e-mailed an information sheet and a link to an anonymous Qualtrics survey, where they were required to provide electronic consent. All participants gave fully informed electronic consent. A convenient time and date for interviewing was then arranged between the participant and researcher (LJ).

At T1, 1 participant refused participation due to lack of available time, and 1 participant failed to respond to 2 separate attempts at e-mail contact, spaced 1 week apart (14% attrition). At T2, 2 participants failed to attend the arranged interview and did not respond to a follow-up e-mail, and 1 participant failed to respond to 2 separate attempts at e-mail contact, spaced 1 week apart (20% attrition).

#### T1 demographics

Participants were aged between 28 and 41 years (*M*_*Age*_ *=* 33.17 years), and infant age ranged from 2 to 13 weeks (*M*_*Age*_ *=* 7.25 weeks). All participants were married. Some participants self-disclosed experiences that were outside the norms of the other recruited participants in this sample, which may have affected psychological experiences during the COVID-19 pandemic: one mother had received private midwifery care, disclosed a history of clinical depression, and was a breastfeeding peer support worker; one mother had experienced a previous stillbirth; one mother had been involved in a clinical trial, designed to provide holistic and tailored support to new mothers; and one mother disclosed a history of clinical anxiety, and was a breastfeeding peer support worker. See Table 1 for T1 demographic information.

**Table 1:** Time one participant demographic information

Participant number	Infant age at time of interview/Weeks	Highest level of education	Occupation	Infant feeding method	Total number of children	County of residence
1	6	A level	Managers, Directors, and Senior Officials	Breastfeeding	1	North Yorkshire
2	10	Doctorate	Professional Occupations	Formula feeding	2	Greater Manchester
3	7	Degree with honours	Professional Occupations	Combination feeding	2	Greater Manchester
4	11	Degree with honours	Managers, Directors, and Senior Officials	Breastfeeding	1	Somerset
5	3	Master’s degree	Managers, Directors, and Senior Officials	Combination feeding	1	Gloucestershire
6	13	Diploma (level unspecified)	Professional Occupations	Breastfeeding	2	West Midlands
7	7	Master’s degree	Professional Occupations	Breastfeeding	2	West Midlands
8	8	Degree with honours	Sales and Customer Service Occupations	Breastfeeding	2	Devon
9	2	Diploma (level unspecified)	Caring, Leisure, and Other Service Occupations	Breastfeeding	3	Suffolk
10	6	Doctorate	Professional Occupations	Breastfeeding	1	Bristol
11	5	Degree with honours	Professional Occupations	Breastfeeding	1	Northamptonshire
12	9	A level	Skilled Trades Occupations	Breastfeeding	3	Lancashire

Occupation categories were taken from the Office for National Statistics [17]. Information regarding UK educational levels were taken from UK Government [18]

#### T2 demographics

Participants were aged between 28 and 41 years (*M*_*Age*_ *=* 34.67 years), and infant age ranged from 6 to 14 weeks (*M*_*Age*_ *=* 10.5 weeks). All participants were married. Some participants self-disclosed experiences that were outside the norms of the other recruited participants in this sample, which may have affected psychological experiences during the COVID-19 pandemic: three mothers had been separated from their infants (for sixty hours, five days, and eight days, respectively) due to unexpected medical complications, and one participant disclosed a history of clinical postpartum depression. See Table 2 for T2 demographic information.

**Table 2:** Time two participant demographic information

Participant number	Infant age at time of interview/Weeks	Highest level of education	Occupation	Infant feeding method	Total number of children	County of residence
13	10	Degree with honours	Professional Occupations	Breastfeeding	3	Durham
14	12	Degree with honours	Professional Occupations	Combination feeding	1	Greater Manchester
15	11	Degree with honours	Managers, Directors, and Senior Officials	Formula feeding	1	Greater London
16	11	Degree with honours	Sales and Customer Service Occupations	Breastfeeding	1	Sussex
17	12	Degree with honours	Professional Occupations	Breastfeeding	1	Cardiff
18	12	Work-based qualifications/National Vocational Qualification (level unspecified)	Sales and Customer Service Occupations	Formula feeding	2	Durham
19	10	Degree with honours	Managers, Directors, and Senior Officials	Combination feeding	1	Merseyside
20	6	Master’s degree	Professional Occupations	Breastfeeding	2	Wrexham
21	9	Degree with honours	Professional Occupations	Breastfeeding	2	Merseyside
22	13	Work-based qualifications/National Vocational Qualification (level unspecified)	Managers, Directors, and Senior Officials	Breastfeeding	1	Wrexham
23	14	Degree with honours	Professional Occupations	Formula feeding	1	Lancashire
24	6	Master’s degree	Not in a Paid Occupation	Formula feeding	3	Durham

Occupation categories were taken from the Office for National Statistics [17]. Information regarding UK educational levels were taken from UK Government [18]

#### Data collection

Before the audio recorded interview commenced, the participant was asked if they had any remaining questions about the study, which were addressed as appropriate. After re-capping topics to be covered during the interview, and re-iterating anonymity, confidentiality, and withdrawal policies; participants were asked if they were happy for the audio recorder to be started. With consent, the audio recorder was started, and the participant was asked to provide verbal consent (by participants confirming they understood and agreed to each of the electronic consent items, spoken aloud by the researcher). All participants gave fully informed verbal consent. Interview schedules were then followed to direct conversation (see supplementary documentation).

After all Interview schedule items had been prompted, the participant was asked if they had anything else to add or if the researcher had forgotten to ask anything before the interview finished. The audio recorder was then stopped, and participants were verbally debriefed. Interviews lasted between 30 and 120 min (*M*_*Time*_ = 53.5 min). Interviews were all conducted by one researcher (LJ), to ensure a standardised approach to data collection. Approximately 1 day after the interview, the participant was e-mailed an electronic copy of the debrief form and was reimbursed £10 for their time. This e-mail was also used to ensure participants were happy with their involvement in the study.

#### Analysis

Audio recordings were transcribed and analysed using NVivo 12 [LJ]. A thematic analysis [19] was employed to elicit the main themes common across both timepoints’ datasets. Thematic analysis follows a methodical, six-step approach where analysts familiarize themselves with the data, generate initial codes, identify themes, review these themes, and finally define and name each theme, before writing-up [19]. Analysis was inductive and consultative [20], meaning all authors were involved in refining the themes, and identifying thematic change across timepoints [21]. Data saturation was judged based on no new concepts being identified through analysis of newly added transcripts [22]. Data saturation was reached after eight (T1) and seven (T2) transcripts, respectively. Participant recruitment continued until twelve participants had been interviewed at each timepoint, evidenced as a minimum requirement for meeting data saturation in qualitative research [23], and to ensure data saturation had been satisfactorily met even if presumed earlier.

A recurrent, cross-sectional approach was taken to analysis, because this form of longitudinal qualitative analysis is evidenced as being most appropriate for use when comparing time points e.g., before and after policy change [24]. Hence, this approach was deemed most appropriate for addressing the study aim, ‘to explore postpartum women’s psychological experiences during different phases of social distancing restrictions imposed in the UK’, so that changes could be captured that were reflective of change. As such, a separate sample of women were recruited at T1 and at T2, so that psychological experiences during the immediate postpartum period (i.e., in the first 3 months after giving birth) could be accurately captured, and so that time-sensitive and specific social, and psychological factors that influence maternal mental health during different phases of the COVID-19 pandemic, could be identified. Recurrent cross-sectional thematic analysis occurs in two stages [24].

To compare psychological experiences of motherhood amidst rapidly changing social distancing guidance, it was deemed necessary by the named authors to use a modified approach to recurrent cross-sectional thematic methodology [24]. Analysis occurred in 2 stages: firstly, independent thematic analyses were conducted for T1 and for T2 separately. Then, comparisons between generated thematic structures were discussed within the context of existing literature [24].

#### Reflexivity statement

The first author is a junior perinatal mental health researcher and experienced the same national lockdown restrictions as the participants, therefore required careful management during data collection and interpretation so not to impose personal views. This was achieved through frequent reflection to ensure identified thematic structure and timepoint comparisons were evidenced by the accounts of participants, achieved by revisiting earlier analysis stages, and through careful review and supervision by an experienced team of mixed methods researchers (VF, JAH, LDP) and a qualitative expert (SAS) throughout the process of data analysis and dissemination. All researchers providing supervision (SAS, VF, JAH, LDP) also have experience in the field of perinatal mental health. The first author also considered their position as a childless researcher as a particular advantage during data collection and analysis, as an element of objectivity (or being the ‘objective outsider’ researcher) was able to be maintained which allowed participants greater ownership of their own unique experiences of parenthood, uninfluenced by suggestion. Finally, careful consideration was also made during study design to ensure the interview schedules were broad and non-leading to allow the participant the flexibility to focus on experiences most salient to them.

## Results

### T1 results

The T1 thematic analysis generated two main themes, each with two sub-themes:

1. Motherhood is Much Like Lockdown

i. Lockdown Exacerbates Postnatal Loss of Independence
ii. Guilt Beyond ‘Normal’ Parenting

2. A Self-Contained Family Unit

i. Lockdown has been a Relief from Social Obligations
ii. Breastfeeding: Triumphs and Tribulations

#### Theme one: motherhood is much like lockdown

All participants at T1 discussed how initial lockdown measures had resulted in lost independence and isolation. These restrictions surpassed the imposed limitations which participants had expected to encounter during the initial postnatal period. Unique financial and informal and formal support stressors were encountered by new mothers, which exacerbated feelings of guilt that were commonly experienced.

#### Lockdown exacerbates postnatal loss of Independence

Some participants likened early postnatal disruption to lockdown disruption, which were both associated with loss of self-identity:

*“Being in lockdown is exactly like being a, being a new mum to a new-born and how it was kind of a bit, it was kind of a double whammy of like restriction on life [laughter]…It’s essentially like lockdown is a bit like being a mother.”* (Participant 3, T1).

Participants in the current study frequently likened lockdown measures with expected postnatal restrictions on leaving the home and associated loss of independence,

*“[lockdown] takes stay-at-home mum to a whole other level.”* (Participant 5, T1).

For others, similarities between lockdown restrictions and the early postnatal lifestyle allowed participants to be largely unaffected by the potential consequences of lockdown restrictions:

*“Having a six-week-old I’m pretty much tied to the house, particularly breastfeeding. Erm so not a lot of places I could’ve gone out anyway without taking her erm you know we’re still, I’m still having to feed her every three hours so it’s not sort of like I can do-do sort of my normal life.”* (Participant 10, T1).

#### Guilt beyond ‘Normal’ parenting

Breaking social distancing guidelines was a source of guilt for new mothers who felt conflicted between needing emotional support and wanting to respect social distancing guidelines:

“*My two best friends and I as well, have been very naughty and met and gone for walks together, because none of us are coping very well mentally…so that has helped tremendously [laughter] erm and I- [Voice wavering] I feel terrible er because I obviously am not trying to do the wrong thing but I think the problem with the way the Government’s handled this is they haven’t been very…I don’t know. It just sounds like they haven’t thought it through very well, which I’m sure they have, but I don’t know. It doesn’t feel like it. It feels like they’ve just done all the wrong things [laughter].”* (Participant 9, T1).

For one mother, financial insecurity prior to accessing the Governmental furlough scheme was a source of intense guilt and distress:

*“I’d wished we hadn’t had [baby] [voice wavering]. Which sounds awful, and it makes me cry. So, it was kind of awful that two weeks after he was born I kind of wished he wasn’t here [crying]. Which is, which is awful to think about… if we hadn’t had [baby] I’d have been sleeping, I’d have been sleeping properly, we wouldn’t’ve had…we wouldn’t have had this financial burden of the credit card and we wouldn’t have been worrying about like - we would’ve had more financial reserves, so… to think back to that now it makes me feel awful…But I…now that things are a bit more certain and he’s smiling now and we’ve got a bit more money coming in and we’ve got the mortgage holiday like it’s all like, I-I- I’m really glad he’s here now.”* (Participant 3, T1).

Also directly related to lockdown measures, lack of structural childcare support was a source of guilt for mothers struggling to manage parenting responsibilities for multiple children:

*“Obviously nursery’s shut as well erm…so it’s been hard because I haven’t had that time [with youngest] that I had with [daughter]. So erm…I guess there’s been quite a lot of guilt that’s probably kicked in, really. Er because I feel like, erm…because [daughter] is so active, as she’s three, I’m always planning activities with her. So, I get the paints out or erm when the days have been nice, we’ve been playing in the garden and I feel like [youngest]‘s always just plonked to the side… I think sometimes it’s been like, probably you’re not doing good enough. Erm, perhaps because the expectations I had pre COVID-19 that it was gonna be this idyllic time [laughter]. So that- that’s been tough, to be honest. Erm and I think, like I said I felt guilty. And a bit of anxiety, really, about not being able to spend as much time [with youngest] as I had done with [daughter]”* (Participant 2, T1).

Receiving paternal support with childcare responsibilities was a release from this guilt:

*“It took a while to work out the system and for both of us to go ‘We don’t have to do this together if we do this together’...and to not feel and to get over the guilt of going just, ‘You have the baby an hour, I need to sleep.’”* (Participant 10, T1).

#### Theme two: a self-contained family unit

Initial lockdown restrictions, including the furlough scheme, had resulted in an increased number of maternal partners working from home [25]. As such, partners were more actively involved in sharing parenting responsibilities than would have been possible pre-pandemic. For mothers at T1, increased paternal involvement and having fewer visitors allowed for: stronger bonds to be established between members of the family unit; more mindful and present adaptation to new parenting roles; and for some, made breastfeeding easier to establish. However, for participants experiencing breastfeeding difficulties, diminished healthcare professional support, discontinued parenting support groups, and school closures were unprecedented stressors which led to early breastfeeding cessation.

#### Lockdown has been a relief from social obligations

Despite lockdown restrictions exacerbating feelings of distress, isolation, and loss of self-identity, eight of the women interviewed also spoke of positive outcomes having arisen from lockdown restrictions. Lockdown restrictions allowed for a more relaxed postnatal period, without experiencing pressures from social obligations:

*“I guess it’s one of the things like my husband was saying as more of a positive to come up is, because we haven’t had any visitors and any pressure on going to see anybody or people coming round, I have just been able to kind of get on with it…he was saying especially with breastfed babies, they normally lose weight but [our baby’s] just been gaining weight ever since she’s been born. I think that’s ‘cause we’ve just been able to just get on with the feeding, especially some days when she’s been like constantly wanting to feed. I haven’t had to worry about people being ‘round or any of that.”* (Participant 11, T1).

Participants spoke of appreciating having the opportunity to settle into their new parenting roles more mindfully:

*“We can literally adapt our lives around the baby’s schedule. So erm without worrying about ‘Oh I’m getting up for work, I haven’t got enough sleep. I’m not going to be able to actually function at work with like only two hours sleep’ so yeah, don’t have to worry about that.”* (Participant 5, T1).

Additionally, many women revelled in receiving additional parenting support from their partners which allowed for the establishment of a routine:

*“It seems to be the same like… everyday, but it’s like it’s good ‘cause like if it was just me on my own like I don’t think I’d manage to get in to a routine. But with husband’s like we’ve gotta stick to it, ‘cause it just keeps you, you know what’s coming-my eldest’s better when he’s got a routine.” (Participant 12, T1).*

Indeed, having additional parenting support from partners was a vital facilitator for maternal emotional wellbeing:

*“My husband’s working from home every day or was, which is great because it means that he can cuddle [baby] for 10 minutes and I can go off and do something for my sanity round the house, even if it’s just hoovering a room. Erm, and, but when he’s at work, which he was yesterday, I find the day’s really, really long.”* (Participant 4, T1).

#### Breastfeeding: triumphs and tribulations

For some participants, restrictions on visitation during the initial lockdown allowed mothers to response-feed their infants easier, and thus breastfeeding was easier to establish:

*“I think even…especially from a breastfeeding point of view it’s kind of like I don’t feel like I have to rush. You know I- there’s nothing to rush for. So, it’s kind of like she’s…she’s gaining weight well.”* (Participant 1, T1).

Additionally, focus was placed on the importance of breastfeeding during a global pandemic due to the immune system protection which breastfeeding provides:

*“It was kind of so that if you do get COVID it’s even more important to feed your baby because, sort of, antibodies and keep your baby healthy and stuff like that.”* (Participant 3, T1).

One participant, who self-disclosed that she was a breastfeeding peer support worker, noted the pivotal role of breastfeeding confidence and healthcare professional support in determining breastfeeding success during the COVID-19 pandemic:

*“Yeah – actually we- so thinking that breastfeeding rates are gonna go up a bit ‘cause people aren’t feeling as much pressure well, ‘cause they can’t go out. So, they’re staying at home and they’re just feeding. However, I think that’ll only be true for people who aren’t really struggling because, like, some of them will hopefully seek help and get it and then be able to persevere. But I think it’ll take quite a high level of resilience and want for those ones who are really struggling, because, yeah, it is-it can be very difficult.”* (Participant 9, T1).

Other mothers found breastfeeding notably more difficult due to the lack of structural healthcare professional support in the early postnatal period:

*“Especially with breastfeeding, there’s nothing better than someone saying, ‘Let me show you’ and, ‘If I hold baby and you do this’…It’s different when you’ve shown someone. When someone-someone talking over the phone, doesn’t make sense at all. Or like you say, the internet you see like 400 different ways of holding a baby [laughter]. So yeah, it’s not always easy on that side of things, definitely.”* (Participant 6, T1).

Additional parenting responsibilities and restrictions on social support in light of schools and breastfeeding support groups being closed was also a noted breastfeeding barrier:

*“I think I’m going to give up breastfeeding probably sooner than I would’ve done…because I’m knackered [laughter] erm…in-in all honesty… just ‘cause I just feel like without the support in the day and without being able to get out and about and see, you know, see my family and stuff, I think it’s…the breastfeeding is getting a little relentless. Erm so yeah I think co- in the sense that, because of, because of lockdown erm- because of COVID, I’m prone to not breastfeed as much.”* (Participant 3, T1).

For such women it was deemed essential to stay kind to yourself,

*“There’s no right or wrong way. You know, at the end of the day the ultimate goal is that my baby needs to be fed. End of. Erm you know, feed him breast milk. Breast milk, er formula. He’s fed. He’s happy. Sweet. That’s done. Job done! You know what I mean? The important thing is actually be kind to yourself, you know?”* (Participant 5, T1).

### T2 results

The Time 2 thematic analysis generated two main themes, each with three sub-themes:

1. Incongruously Held Views of COVID-19
  i. Frustrated by Lockdown Restraints on ‘Normal’ Life
  ii. Mums have Slipped Through the Net
  iii. The Pandemic Isn’t All Bad
2. Mothering amidst the pandemic
  i. Guilt, Inadequacy, & Anxiety
  ii. Assessing Risk & Breaking Guidelines
  iii. Non-existent Breastfeeding Support

#### Theme one: incongruously held views of COVID-19

All twelve participants at T2 expressed exhaustion with imposed restrictions on personal freedoms, whilst also acknowledging positive outcomes derived from the COVID-19 lockdown. Participants were disappointed by their postnatal experiences, which were constrained by social distancing restrictions. Postnatal experiences amidst the COVID-19 pandemic were at odds with the socially connected and bustling experiences that they had envisioned for themselves during pregnancy. Participants made suggestions for more active efforts to be made to help support new mothers during the pandemic at national and structural levels, including: campaigning to educate new mothers on where to access formal support; continuing or re-instating routine infant health checks; and allowing partners to be present throughout all stages of labour. Positive outcomes which were derived from lockdown restrictions concerned having fewer visitors in the early postnatal period, which allowed mothers and maternal partners to be more present and mindful in adapting to new parenting roles, allowed greater paternal involvement in the family unit, and made breastfeeding easier to establish.

#### Frustrated by lockdown restraints on ‘Normal’ life

For women at T2, there was a great sense of frustration around not being able to lead a normal postnatal lifestyle:

*“I felt like I’d been imprisoned. I was just, like, sick and tired of being in this living room. I think that was really hard.”* (Participant 15, T2).

Many participants reported struggling with the long-term effects of social isolation and restrictions on independence:

*“I’m getting to the point now where; do you know what? I want my mum to be able to give her granddaughter a cuddle. I want to be, you know, this coming lockdown, I want to be able to up and sit at my mum’s and have a comfortable place to feed and a cup of tea while I feed.”* (Participant 19, T2).

There was a general sense that COVID-19 and associated restrictions had exacerbated early postnatal challenges. Consequentially, some participants expressed a desire for a Governmental response to extend maternity leave for those affected by COVID-19:

*“I dunno whether maybe it would help if they looked at sort of extending people who had their babies like at the start of lockdown or even whenever, maybe extending some maternity leave, that kind of thing. So, we can do certain things like the classes and stuff. Like maybe, I mean like just a couple of months but I feel like now, whereas I’ve missed out on like a few months where I could be out doing those things, like, it’s just been expected for me to even like just get on with it now or take unpaid leave, which I can’t really afford to do.”* (Participant 17, T2).

Other frustrations regarding COVID-19 restrictions included the closure of parenting support groups. Many participants expressed a desire for support groups to be reinstated:

*“There’s nothing like just meeting people or, you know, just naturally building friendships when you go to baby groups and things. There was people that you’d see at every group and you’d just start to get, you know, become friends because you were always there and things so erm, just- especially some people would really struggle probably to meet new people, anyway. So erm that must be really challenging… It’d be great if they could start [baby groups] again, especially for non-mobile babies ‘cause you could do the social distancing.”* (Participant 21, T2).

These critiques were mentioned in relation to injustices compared with economic-building, non-essential services:

*“I signed a petition for baby groups, basically. If we can go to pubs then we can have our baby groups, it’s just insane. It’s not, ‘cause it’s economy versus social.”* (Participant 22, T2).

#### Mums have slipped through the net

Many of the participants acknowledged that the perinatal period is a particularly vulnerable and disruptive time in a woman’s life, and expressed that they would have liked a more active Governmental response to informing new mothers of where to find support during this difficult time:

*“I think they could’ve done a campaign, just something to say, you know, we know it’s hard for you, we’re here. This is where you can go. This is what you can do…I think there are mums out there who do feel lonely, and they don’t know where to go.”* (Participant 15, T2).

Criticisms were also aired regarding insufficient structural support, and concerns were raised regarding the potential consequences of such disrupted support:

*“I think they could’ve thought a lot more clearly about the support, new mums-in terms of checking that emotionally they’re okay and also checking their home environment. I actually think they’ve just kind of stopped it without really thinking about the implications of that and I think that, you know, they should’ve continued home visits in a relatively safe way, and yeah, having a proper six week check with the GP I think was important and more ways of checking on the health, you know, the progress of your baby. I just think having such a long period of time without any contact or any checks on the baby at all is dangerous [laughter]”* (Participant 20, T2).

There was a consensus that women had been overlooked and marginalised by the Governmental response to the COVID-19 pandemic, which left women feeling abandoned and alone:

*“I do feel like we’ve been let down, to be honest. I do feel like we’ve been let down. That and new mums, ‘cause even from a safeguarding perspective, it’s just not fair, that support. I don’t really understand why it’s not [sigh] yeah, why it’s not been prioritised to this extent.”* (Participant 16, T2).

Other criticisms included having a desire for social support bubbles to have been established for new parents earlier in pandemic restrictions:

*“I understand what-why the rules are what they were, but if I could’ve, you know, like they’ve done support bubbles for single parents and anyone that’s on their own? I suppose in those early days I could’ve done with support from family.”* (Participant 21, T2).

A final suggestion made by participants to improve the support available to new mothers during the early postnatal period included greater provisions to be put in place for allowing partners to be present during labour:

*“I think they should’ve put tests…as soon as possible they could’ve put testing in place for partners, that are women who are in labour, so the partner can come in… to be on your own in labour is horrendous. It’s just…it’s, that’s-nobody should have to go through that, I don’t think.”* (Participant 22, T2).

#### The pandemic Isn’t all bad

Despite frustrations and setbacks caused by COVID-19 restrictions, all interviewees expressed that there had been some positive outcomes which had arisen from imposed restrictions. Many interviewees talked about feeling less pressure from visitors and having the unique opportunity to be more mindful and to relax into their new parenting roles:

*“I could just recover on my own [laughter] just recover in the house. Didn’t have to change out my pyjamas, I didn’t have to have the house clean…and have to put up a front and to see guests. So, I had that time really, alone.”* (Participant 14, T2).

Additionally, mothers appreciated having the opportunity to establish a clear and responsive routine with their infants, without disruption from visitors:

*“It’s enabled us to instil a routine with her. So, she’s a great sleeper now and a happy baby and we-because you don’t get that stream of visitors.”* (Participant 15, T2).

For other mothers, restrictions on between household mixing had given the unique opportunity for partners to be more inclusively involved in the family unit:

*“It was lovely to-when we came home from the hospital; it was just me, my husband, my little girl and the baby. It was just the 4 of us, and it gave [eldest] chance to get used to the baby before other people started coming round, kinda thing.”* (Participant 18, T2).

Breastfeeding practice was reportedly easier for some mothers due to reduced visitations, allowing women to more easily persevere and response-feed their babies:

*“One good thing about lockdown is that had all the time in the world to be able to breastfeed, get it right and erm yeah because I wasn’t doing anything else.”* (Participant 24, T2).

Having one’s partner at home was an invaluable support for sharing parenting responsibilities, which allowed women to persist with breastfeeding practice:

*“I’ve got the baby to feed so often and just been, you know, sat there doing nothing else but feed the baby at times so [husband] does everything else. If he wasn’t there, going back to work and things like that when the kids are back to school- there’d’ve been no way for me to carry on [with breastfeeding] ‘cause it was taking so much of my time.”* (Participant 13, T2).

However, there was also a consensus among participants that the unanticipated benefits caused by imposed social distancing restrictions did not outweigh the unprecedented difficulties of transitioning to motherhood during a pandemic:

*“You know people want to come round and see the new baby and that’s great and it’s really lovely but I do think probably could be quite overwhelming for a new mum…So those things I kinda feel like, yeah, they’ve been really great and has definitely benefited from that…but you know, I still don’t think those positives outweigh sort of the negatives- I wouldn’t choose for it to be that way, let’s say, because there’s so many things that I feel like we’ve missed out on in terms of you know him [baby] meeting his family.”* (Participant 16, T2).

#### Theme two: mothering amidst the pandemic

COVID-19 posed extraordinary stressors for new mothers, which exacerbated feelings of guilt. Sources of guilt included judgement from others for taking their infant out in public amidst the pandemic and feeling a burden for attending hospital appointments. Mothers also frequently mentioned anxieties concerning the easing of lockdown restrictions. Such anxieties were, at times, disruptive to daily functioning. Mothers at T2 also experienced notable breastfeeding barriers due to lack of face-to-face support from healthcare professionals and peers. For some, this unfortunately contributed towards early breastfeeding cessation.

#### Guilt, inadequacy, & anxiety

Many interviewees noted that the early postnatal period was a particularly vulnerable time for the experience of guilt. COVID-19 exacerbated feelings of guilt due to experiences of judgement from strangers regarding taking one’s infant outside during the pandemic:

*“I went on the train and went to M&S with [baby] and someone was like ‘I can’t believe you’ve brought her out here’ and I’m like well, okay, sorry.”* (Participant 15, T2).

Other sources of guilt included being made to feel a burden for attending hospital appointments:

*“I felt almost guilty for going to appointments, I didn’t feel guilty, but I was made to feel kind of like I shouldn’t be there and it was all of a bit of an extra inconvenience, and that wasn’t erm that wasn’t a good way to feel.”* (Participant 20, T2).

All participants expressed concerns about easing social distancing measures. Some participants were extremely anxious of the prospect of going out in public and were following guidelines stringently:

*“We were shielding so we weren’t able to go anywhere at all, really. I literally stayed in the house all the time apart from going to my antenatal appointments, which weren’t very often.”* (Participant 13, T2).

For some mothers, COVID-19 related anxieties became disruptive to one’s ability to function in day-today activities:

*“I never had anxiety before…I never, I’ve never suffered with it. But even if I like, if I see [baby]- if I go to like if I pop to the supermarket now, I get all like panicky. I feel like I’ve got to rush in and rush out as quickly as I can. I dunno if it’s ‘cause it’s just odd when you go in there now and the one-way systems and all like being a bit weird or I don’t know if I would’ve been like that anyway because I’m away from the baby, but I suppose I wouldn’t’ve overthought it before.”* (Participant 17, T2).

#### Assessing Risk & Breaking Guidelines

All participants talked about rationalising the breaking of social distancing restrictions, within the context of weighing up the perceived costs of limited social interaction with the perceived benefits of receiving much needed support during the immediate postnatal period:

*“I think that’s what a lot of mums are doing behind closed doors, sort of weighing up…you’ve gotta weigh up risks, a lot of people at this point now. It’s just like erm some people have moved in with their mums.”* (Participant 15, T2).

At T2, flexibility was expressed regarding social distancing compliance, with mental health concerns being a major consideration when weighing up perceived benefits of breaking guidelines:

*“I snuck my mum in ‘cause when she had her operation, it just got too much. I needed someone. So, she came and stayed for a week. It was a bit of a risk-based decision, really, because we’d been self-isolating, so I knew we were fine. She’d had a COVID test ‘cause of that surgery so I knew she was fine.”* (Participant 22, T2).

Despite increased comfort with breaking social distancing restrictions at T2, moral conflict was still expressed concerning the opposing motivations to protect loved ones from the dangers of COVID-19 and missing receiving support and intimacy:

*“You’re constantly mindful that [grandparents are] still a risk and a danger and you certainly don’t wanna put family members at risk. At the same time, you kinda feel like you need the support, they want to give it and they want to see their new grandchild so it…yeah…it’s difficult to kind of get that balance.”* (Participant 20, T2).

#### Non-existent breastfeeding support

For breastfeeding mothers, lack of face-to-face contact with healthcare professionals was perceived as a significant barrier to establishing breastfeeding, especially regarding concerns about infant weight gain:

*“I was paralysed that I wasn’t doing it [breastfeeding] right and I was terrified that [baby] was gonna lose weight again. So, when I… I think I rang the health visitor in the end and was like, can I… can you weigh him? ‘Cause I don’t know if he’s losing weight or whatever, and that must’ve been 3 days after we got home. So, she-I was like in tears down the phone, she erm got me to go to her office and she was all like PPE-ed up and then they did weigh him. Then, like, I’ve not been since.”* (Participant 17, T2).

Lack of breastfeeding support groups allowing for peer support and encouragement was also a COVID-19 impacted barrier to breastfeeding,

*“I wanted to breastfeed this baby, that’s gone downhill, and that doesn’t help because of the, like, no support groups because of COVID and everything. I know everything’s online and everything’s…there’s all the video stuff but to have a video in the right place where you’re feeding baby or trying to- or them, seeing what baby’s doing and stuff, it’s very difficult. So, for feeding-wise that’s yeah, that’s been messed up with COVID, really, I think.”* (Participant 24, T2).

For some women, such insufficient support led to early breastfeeding cessation:

*“Initially was trying to breastfeed erm but I just couldn’t do it, and then I think because I didn’t have that support there to help with that erm and I was worried I wasn’t getting anything, I moved on to formula feeding so we just bottle feed now.”* (Participant 23, T2).

## Discussion

The current study used a recurrent, cross-sectional approach to thematic analysis to explore women’s psychological experiences during the COVID-19 pandemic. Major themes identified at T1 were, ‘*Motherhood is much like lockdown*’ and ‘*A self-contained family unit* ‘. Each major theme contained two sub-themes. Major themes identified at T2 were, ‘*Incongruously held views of COVID-19*’ and ‘*Mothering amidst the pandemic*’. Each major theme contained three sub-themes.

Women at both T1 and T2 disclosed feeling guilty, which had been worsened by COVID-19. Reasons given for experiencing guilt included breaking social distancing guidelines due to mental health concerns (T1), and perceived judgement from others for taking one’s infant outside during the pandemic (T2). Guilt was also experienced in relation to negative thoughts being held about parenthood, due to difficulties coping with lost childcare support and experiencing financial strain due to COVID-19 (T1). The impact of COVID-19 on mental health has been more impactful for parents, and strongly related to increased financial insecurity and home-schooling responsibilities [26]. Implementation of the furlough scheme in the UK for those unable to work from home has been effective in preventing risk of developing a common mental health disorder [27], which may explain the absence of financial strain among T2 accounts. Given current study findings, national prioritisation of schools re-opening may also have beneficial outcomes for parental emotional wellbeing [28].

Concerns were expressed regarding maternal and infant safety (T2). Additionally, breastfeeding was discussed as being more difficult for women who were already struggling with breastfeeding difficulties at both time points. This was attributed to reduced face-to-face health visitation (T1) and breastfeeding support (T2). Other qualitative work into maternity care has also identified maternal concerns regarding maternal and infant safety due to the limitation of face-to-face health checks [29]. These findings are concerning, as social distancing restrictions appear to have exacerbated physical and emotional risks for mothers and infants at the greatest need of support. Virtual care is of limited effectiveness in supporting new mothers, compared with face-to-face healthcare [14, 29]. Evidence therefore recommends a revised policy that prioritises face-to-face healthcare visitation.

Women at both T1 and T2 valued the unique opportunity to receive additional paternal support with childcare, which they would not have received without COVID-19 imposed social distancing restrictions. Current UK legislation provides fathers with the rights to one to 2 weeks of paid paternity leave, or 37 weeks leave when shared with maternal leave [30]. Current findings suggest that the latter approach to providing parental leave may have the potential to improve breastfeeding outcomes, strengthen family relationships, and improve paternal interaction quality with infants [31].

Furthermore, participants at both timepoints found the transition to parenthood easier as a consequence of having fewer visitors, in light of social distancing restrictions. For some participants, breastfeeding was found to be particularly easier to establish and maintain. This supports previous research demonstrating that breastfeeding support needs to be appropriately timed, positive, and non-judgemental in order to have a positive impact on breastfeeding practice [32]. Additionally, previous research suggests that breastfeeding practice can also be adversely affected if family’s views do not well coincide with parental infant feeding preferences [33]. Thus, the pandemic may have allowed mothers the unique opportunity to take greater autonomy over their infant feeding method. However, there were harmonious feelings among T2 participants that although having fewer visitors is beneficial for those not experiencing breastfeeding challenges, that losing access to informal and formal sources of breastfeeding support exacerbates difficulties and increases risk of early breastfeeding cessation for those experiencing breastfeeding challenges [34]. As a result, causing women who are most in need of using breastfeeding support services to ‘fall through the cracks’. Overall, although women were able to take some positives from their experiences of parenthood during the pandemic, accounts were overwhelmingly negative in their focus on inaccessibility to informal and formal sources of support, suggesting attempts to ‘make the best of a bad situation’ [35]. Given these findings, future research should also seek to explore factors which may contribute toward adopting this more resilient mindset during the pandemic, among new mothers.

Participants at T1 perceived lockdown disruption to be an extension of already present challenges of transitioning into new motherhood. This contrasted starkly with the experiences of mothers at T2, who reported exhaustion with restrictions placed on access to support that were beyond commonly experienced challenges related to new parenthood. T2 participants were also frustrated with the inability to share parenthood milestones with friends and family, which was a source of sadness and guilt for participants at T1. Recent literature which has examined the emotional impacts of COVID-19 among parents, has identified high prevalence of parenting exhaustion [36]. Lockdown restrictions on access to social and structural support have resulted in an increase in parental demand, due to chronic exposure to parenting stress [37]. This is problematic as parental burnout adversely affects parent-child interactions and maternal emotional wellbeing [38]. Participants at T1 and T2 believed that re-establishing parenting groups should be prioritised as lockdown restrictions ease, especially for immobile infants whereby social distancing measures could be more easily adhered to. Indeed, prioritisation of parenting group re-establishment would further ensure that mothers have adequate access to emotional support and thus facilitate positive emotional wellbeing and breastfeeding outcomes during this, and similar crises.

Mothers at T1 expressed much moral conflict between wanting to adhere with social distancing guidelines and needing emotional support, while T2 participants frequently made risk-based decisions to gain social support. These decisions were reinforced by frustrations held about lockdown restrictions on personal freedoms. Rationalisation of breaking guidelines may be explained by cost-benefit analysis [39]. Extended periods of social isolation led to greater negative affect i.e., an increase in perceived barriers to maintaining adherence with social distancing guidelines, which may in turn increase the perceived benefits of breaking guidelines [40]. Pregnancy and the early postnatal period are a time of heightened risk of experiencing emotional distress [12], and mothers at both T1 and T2 found prolonged isolation from friends and family to be particularly strenuous. Current findings evidence policy recommendations to improve accessibility to support for new mothers i.e., allowing mothers to bubble with a primary support partner e.g., maternal grandmother, with an aim to improve compliance with social distancing restrictions.

T2 participants frequently mentioned there needing to be prioritisation of paternal support throughout birth, not just during active labour. This may have not been salient in T1 accounts due to more heterogenous experiences of birth at this timepoint: three participants had given birth before social distancing restrictions were implemented, one participant had had a home birth, and two participants had been allowed partners to be present at some hospital appointments. Although birth is a positive experience for most mothers [41], it can also be traumatic and disempowering [42]. Lack of paternal involvement in pregnancy has been linked with increased risk of premature birth and low infant birth weight [43]. Although well-intentioned, implemented restrictions on maternal support during pregnancy and labour [1, 44] have appeared to have had a negative impact on maternal emotional health. Recommendations are thus made to allow mothers one support partner for all necessary hospital appointments and home visits, not solely during active labour and during the immediate postnatal period.

### Strengths, limitations, and future directions

Recurrent cross-sectional thematic analysis [19, 21, 24] allowed for directionality to be established. Similar themes were identified across both timepoints, with increased exhaustion related to parenting and social distancing restrictions at T2. This provides strong evidence that COVID-19 lockdown restrictions have had an adverse cumulative effect on maternal mental health. Howbeit, using a modified analytical approach was an identified limitation of the current study, deviating from reported procedures in previously published perinatal literature whereby repeat interviewing had been reported [45]. This is problematic because lack of homogenous sampling at independent timepoints may have unintentionally compromised methodological rigour. Although problematic, recruiting separate samples of women for T1 and T2 interviews for the purpose of the current study allowed for the necessary flexibility to identify time-sensitive and specific social and psychological factors, which were influential determinants of maternal emotional wellbeing, to be identified during different phases of imposed national lockdown restrictions in the UK.

In recent quantitative literature, clinical cut-offs for postpartum anxiety and depression symptoms were concerningly high when compared with national prevalence of self-reported clinical diagnoses [15]. Current findings offer additional insights by identifying structural, social, and psychological factors which may have contributed towards elevated levels of clinically relevant depression and anxiety [15]. Study findings highlight important considerations for policy and practice to improve accessibility to support during this, and similar crises. The use of telephone and video calling for interviewing allowed nationwide participation. This allowed for a diverse representation of women from different counties to be included in analysis: improving the transferability of findings. Data collection was in rapid response to imposed COVID-19 restrictions and subsequent changes to social distancing guidelines [6]. This allowed for valid exploration of the effects that changing social distancing guidelines had had on psychosocial experiences of motherhood.

There were limitations worth consideration in this study, regarding transferability of findings. Heterogeneity of some participant characteristics may have confounded findings. The following additional psychological stressors that were self-disclosed may have increased the likelihood of experiencing emotional distress: history of clinical anxiety (1 participant), history of clinical depression (1 participant), separation from infant immediately after birth due to medical complication (3 participants) and having experienced a previous still birth (1 participant). Some participants self-disclosed unanticipated, potentially protective factors against emotional distress: being involved in a clinical trial designed to provide holistic and tailored support to new mothers (1 participant), receiving private healthcare professional support (1 participant), and being a breastfeeding peer support worker (2 participants). Thus, some caution must be taken regarding transferability of findings, however, this also provides excellent bases upon which more nuanced investigations can be designed and conducted, with specific foci on groups which were less represented in the current study (e.g., those with diagnosed mental illness, those from minority ethnic backgrounds, those who accessed private healthcare).

## Conclusion

The current study used recurrent cross-sectional thematic analysis to explore psychological experiences of motherhood during the COVID-19 pandemic. Current findings reveal that prolonged periods of enforced social distancing restrictions have had a detrimental effect on maternal mental health. Implementation of the furlough scheme in the UK was particularly effective in alleviating feelings of anxiety and guilt, and current findings suggest that prioritisation of schools re-opening would also alleviate parenting strain and exhaustion. Recommendations are made regarding social distancing policy to support maternal wellbeing more effectively: prioritisation of re-establishing parenting groups as lockdown restrictions ease, allowing mothers to bubble with a primary support partner during national lockdown e.g., maternal grandmother, and allowing mothers one support partner for all necessary hospital appointments and home visits, not solely during active labour and during the immediate postnatal period. Suggestions are also made for methods of improving accessibility to support for new mothers during this, and similar crises, at a community level: providing informational resources whereby mothers can access up to date and consolidated perinatal guidance based on updates from daily governmental briefings and prioritising face-to-face breastfeeding support from healthcare professionals.

## Supplementary Information

**Additional file 1: Supplementary file 1.** Postnatal interview schedule, timepoint 1. Interview schedule developed for all conducted timepoint 1 interviews. Interview schedule was developed in collaboration with all named members of the research team and aimed to explore the psychological experiences of UK women: before hearing about COVID-19, now [since the start of social distancing restrictions being imposed in the UK on the 23 March 2020], thinking about the future, and thinking about their general thoughts and opinions of COVID-19.

**Additional file 2: Supplementary file 2.** Postnatal interview schedule, timepoint 2. Interview schedule developed for all conducted timepoint 2 interviews. Interview schedule was developed in collaboration with all named members of the research team and aimed to explore the psychological experiences of UK women: at the start of COVID-19 restrictions being imposed in the UK [23 March 2020], since the initial easing of COVID-19 restrictions [11th May 2020], thinking about the future, and thinking about their general thoughts and opinions of COVID-19.

## Data Availability

The datasets used and/or analysed during the current study are part of a common dataset from The PRaM Study. The datasets are not publicly available due to the sensitive nature of the interviews however they are available upon reasonable request from the PRaM Chief Investigator (Dr. Victoria Fallon) at: V.Fallon@liverpool.ac.uk.

## Abbreviations

PHE: Public Health England
RCOG: Royal College of Obstetricians and Gynaecologists
SARS-CoV-2: Severe Acute Respiratory Syndrome Coronavirus 2 (a.k.a. COVID-19)
UK: United Kingdom
WHO: World Health Organization
