# Supplementary material for: Postpartum women’s psychological experiences during the COVID-19 pandemic: a modified recurrent cross-sectional thematic analysis
Source: BMC Pregnancy Childbirth. 2021 Sep 17;21:625. doi: 10.1186/s12884-021-04071-2 (PMC8445650; doi:10.1186/s12884-021-04071-2)
Supplement: Supplementary file 1 — Additional file 1: Supplementary file 1. Postnatal interview schedule, timepoint 1. Interview schedule developed for all conducted timepoint 1 interviews. Interview schedule was developed in collaboration with all named members of the research team and aimed to explore the psychological experiences of UK women: before hearing about COVID-19, now [since the start of social distancing restrictions being imposed in the UK on the 23 March 2020], thinking about the future, and thinking about their general thoughts and opinions of COVID-19. [file 12884_2021_4071_MOESM1_ESM.docx]

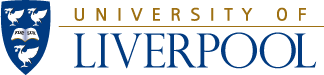


***Postnatal topic guide, timepoint 1***

Thank you for agreeing to talk about your experience of the Covid-19 pandemic at the start of social distancing measures being implemented during the COVID-19 outbreak. We are interested in your own personal experience of new life with a baby at the start of social distancing measures being implemented during the COVID-19 pandemic, which may be different from other people. We would like to record the conversation with your permission. We will be able to arrange an opportunity for you to see the transcript. Should you wish to stop the interview at any time, or take a break, please tell me. Also you do not have to answer any question(s) which you do not feel comfortable answers. Our discussions will remain confidential and all data will be anonymised (see information sheet).

I would like to ask you some more open questions about your experiences of the current pandemic, regardless of whether you have been unwell or not. The interview is structured in four parts: we will ask you some information about how new motherhood was before we knew about Covid-19, how motherhood is for you currently (at the start of social distancing measures being implemented); what you think the future might hold; and finally some general questions about media, social media, what the government could do, and what advice you would give to other new mothers.

Thank you for agreeing to speak to me today about your experience. Before we begin, can you provide me with your initials and the day and month of your birthday. This is so we can match your consent in this interview with your electronic consent and no other use will be made of this information.

1. What is your age?
2. How old is your youngest baby (in weeks)?
3. What is your highest education level (if currently enrolled, mark the previous grade or highest degree received)?

*Response options:*

Completed postgraduate education (Master’s degree/PhD or equivalent)

Completed undergraduate education (degree or equivalent)

Completed A levels (or equivalent)

Completed GCSE’s (or equivalent)

No qualifications completed

Other qualification(s) (please specify)

1. What is your occupation (if you are currently on maternity leave, this includes your occupation before you started maternity leave)?

Response options:

Managers, directors, and senior officials

Professional occupations

Associate professional and technical occupations

Administrative and secretarial occupations

Skilled trades occupations

Caring, leisure, and other service occupations

Sales and customer service occupations

Process, plant, and machine operatives

Elementary occupations

Not in a paid occupation

1. Which NHS Trust is providing your postpartum care?

A. Before Covid-19

Can you tell me what motherhood was like before you heard about COVID 19/coronavirus:

What did you do?

With family and friends

Activities: e.g. parenting classes, support groups, hospital appointments, home visits from healthcare professionals etc.

How was your general health?

How did you feel?

How was your mood?

What things made you anxious, lonely or sad?

What things made you happy?

How did you cope?

How was new life with baby?

How did you feel about motherhood?

What support did you receive as a new mother?

How were you feeding your baby? Contributing factors/feelings?

What advice/information did you receive about motherhood?

What advice/information did you receive about infant feeding?

B. At the present time

Can you tell me about how things are now?

What are you doing?

With family and friends

Activities: e.g. parenting classes, support groups, hospital appointments etc.

How is your health?

How have these changed?

What are you no longer doing?

Are you doing anything new?

How do you feel?

How is your mood?

What things make you anxious, lonely or sad?

What things make you happy?

How are you coping?

How do you feel about motherhood now?

How has this changed new life with baby?

How has this changed the support you receive as a new mother?

How has feeding your baby changed?

What advice/information did you receive about COVID-19 in terms of life with a baby?

What advice/information did you receive about COVID-19 in terms of infant feeding?

C. The future?

What do you think you'll be doing in the future?

Activities: e.g. parenting classes, support groups, hospital appointments etc.

How might your activities have changed?

What might you no longer be doing?

What new things might you be doing?

How do you imagine your health might be?

How do you think you might feel?

How might your mood be?

What things do you think may make you anxious, lonely or sad?

What things might make you happy?

How might you be you coping?

How do you think this may change your experience of new motherhood in the future?

How do you think this may change the support you receive in the future?

What advice/information would you like about COVID-19 in terms of life with a new baby in the future?

How do you think your relationship with your baby might be in the future? Contributing factors?

What advice/information would you like about COVID-19 in terms of infant feeding in the future?

How might COVID-19 change your feelings about new motherhood in the future?

How might this change your infant feeding practice in the future?

D. We are also interested in your general views

What do you think about the way people are talking about the virus?

Media, social media, communities, friends and neighbours?

Other mothers, health professionals?

Have there been any acts of unkindness that you have experienced or seen happen to others, related to motherhood?

How does that make you feel?

Are there any acts of kindness that you have experienced or seen happen to others, related to motherhood?

How does that make you feel?

Have there been any acts of unkindness that you have experiences or seen happen to others, related to infant feeding?

How does that make you feel?

Are there any acts of kindness that you have experienced or seen happen to others, related to infant feeding?

How does that make you feel?

What would you like local government, health services, and/or government to do for you as a new mother?

Medical needs?

Mental wellbeing needs?

Support?

Infant feeding needs?

What advice would you give to other mothers in the same situation?

Before we finish this interview, is there anything else you would like to talk about, or anything that I have forgotten to ask about?

Thank you for your time. We will leave you with a list of organisations that you can contact if you need support.
