# Supplementary material for: Postpartum women’s psychological experiences during the COVID-19 pandemic: a modified recurrent cross-sectional thematic analysis
Source: BMC Pregnancy Childbirth. 2021 Sep 17;21:625. doi: 10.1186/s12884-021-04071-2 (PMC8445650; doi:10.1186/s12884-021-04071-2)
Supplement: Supplementary file 2 — Additional file 2: Supplementary file 2. Postnatal interview schedule, timepoint 2. Interview schedule developed for all conducted timepoint 2 interviews. Interview schedule was developed in collaboration with all named members of the research team and aimed to explore the psychological experiences of UK women: at the start of COVID-19 restrictions being imposed in the UK [23 March 2020], since the initial easing of COVID-19 restrictions [11th May 2020], thinking about the future, and thinking about their general thoughts and opinions of COVID-19. [file 12884_2021_4071_MOESM2_ESM.docx]

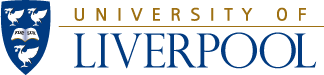


***Postnatal topic guide, timepoint 2***

Thank you for agreeing to talk to me today about your experience of motherhood during the COVID-19 pandemic.

We are interested in your own personal experience, which may be different from other people – so there are no right or wrong answers and you will not be judged based on what you say.

We would like to record the conversation with your permission.

We will be able to arrange an opportunity for you to see the transcript.

Should you wish to stop the interview at any time, or take a break, please tell me.

Also you do not have to answer any question(s) which you do not feel comfortable answering.

Our discussions will remain confidential and all data will be anonymised (see information sheet).

The interview is structured in four parts:

How life was before we knew about Covid-19

How life was at the start of lockdown restrictions (as of the 23^rd^ March 2020)

How motherhood is for you now since the easing of lockdown restrictions, as of the 13th May 2020.

Your opinion on media, social media, and government dealing with the pandemic, and what advice you would give to other new mothers.

[verbal consent obtained]

1. What is your age?
2. How old is your youngest baby (in weeks)?
3. What is your highest education level (if currently enrolled, mark the previous grade or highest degree received)?

*Response options:*

Completed postgraduate education (Master’s degree/PhD or equivalent)

Completed undergraduate education (degree or equivalent)

Completed A levels (or equivalent)

Completed GCSE’s (or equivalent)

No qualifications completed

Other qualification(s) (please specify)

1. What is your occupation (if you are currently on maternity leave, this includes your occupation before you started maternity leave)?

Response options:

Managers, directors, and senior officials

Professional occupations

Associate professional and technical occupations

Administrative and secretarial occupations

Skilled trades occupations

Caring, leisure, and other service occupations

Sales and customer service occupations

Process, plant, and machine operatives

Elementary occupations

Not in a paid occupation

1. Which NHS Trust is providing your postpartum care?

A. Before Covid-19

Can you tell me how life was before you heard about COVID 19/coronavirus:

What did you do?

With family and friends

Activities: e.g. antenatal classes, support groups, hospital appointments, home visits from healthcare professionals etc.

How did you feel?

What things made you anxious, lonely or sad?

What things made you happy?

How did you imagine new life with a baby would be?

How was your experience of maternity care?

Access to routine care?

Access to support?

Relationships with midwives?

What advice/information did you receive about motherhood?

What advice/information did you receive about infant feeding?

B. Start of lockdown (23^rd^ March 2020)

Can you tell me how life was immediately after lockdown on the 23^rd^ March 2020?

What did you do?

With family and friends

Activities e.g. antenatal classes, support groups, hospital appointments, home visits from healthcare professionals etc.

How did you find feeding your baby?

Was your mood affected by lockdown restrictions?

What things made you anxious, lonely, or sad?

What things made you happy?

How did you imagine new life with a baby would be?

Was your experience of maternity care affected by lockdown restrictions?

What was good about the care you received?

What was bad about the care you received?

Access to routine care?

Access to support?

Relationships with midwives?

What advice/information did you receive about motherhood?

What advice/information did you receive about infant feeding?

Can you tell me about your birth experience?

- If anything, can you tell me what changed about your birth plan due to initial lockdown restrictions on the 23^rd^ March 2020?

- If anything, can you tell me what changed about your birth experience due to initial lockdown restrictions on the 23^rd^ March 2020?

The next section of this interview will focus on your experience of life with a new baby since the 13th May 2020 (when the UK Governmental announced easing lockdown restrictions, such as: unlimited socially distanced exercise, access to parks and open spaces, encouragement to return to work if you are able to and it is safe to do so, and outside gatherings of up to 6 people outside of your household) during the COVID-19 pandemic.

C. At the present time

Can you tell me about how things are now, since the easing of lockdown restrictions as of the 13th May 2020?

What are you doing?

With family and friends

Activities: e.g. parenting classes, support groups, hospital appointments etc.

What is positive/negative about being a mother?

What support do you receive as a new mother?

How does the support meet your needs – what else was needed?

How does motherhood compare to how you thought it would be? Why/how?

If at all, has this changed since the start of lockdown?

How do you feel?

Have the easing of lockdown restrictions, as of the 13^th^ March 2020, affected your mood?

What things make you anxious, lonely or sad?

What things make you happy?

If at all, has this changed since the start of lockdown?

How are you coping/what coping strategies are you using during COVID?

Why did you use these strategies?

How effective were the strategies?

Who is benefitting from you using these strategies and how are these strategies being of benefit?

Do you plan on continuing to use these strategies?

If at all, how have these strategies changed since the start of lockdown?

What or how have you changed as a result of COVID-19?

How have your relationships with your children, family, friends changed?

How have your relationships with midwives/health visitors changed?

Do you feel differently about how you would cope with future/similar events such as COVID-19, and if so how?

What changes have you made to your work, or role, as a result of COVID-19?

What new opportunities, or ventures have you been involved in as a result of COVID-19?

How have your beliefs and values about you or your life changed as a result of COVID-19?

If at all, how have these experiences changed since the start of lockdown?

How do you feel about motherhood now?

How has COVID-19 changed new life with baby?

How has the COVID-19 outbreak affected the support you receive as a new mother?

Have you received any advice/information about COVID-19 in terms of life with a baby? If yes, what advice/information have you received?

Have you received any advice/information about COVID-19 in terms of infant feeding? If yes, what advice/information have you received?

How has the COVID-19 outbreak affected how you feed your baby?

If at all, how have these experiences changed since the start of lockdown?

Have there been any acts of kindness/unkindness that you have experienced or seen happen to others, related to caring for a baby?

If at all, how has this changed since the start of lockdown?

Have there been any acts of kindness/unkindness that you have experienced or seen happen to others, related to infant feeding?

If at all, how has this changed since the start of lockdown?

D. Information/support needs?

What advice/information would you have liked to have received about COVID-19 in terms of life with a new baby?

What advice/information would you have liked to have received about COVID-19 in terms of infant feeding?

What would you like local government, health services, and/or government to do for you as a new mother?

Medical needs?

Mental wellbeing needs?

Support?

Infant feeding needs?

What advice would you give to other mothers in the same situation?

Before we finish this interview, is there anything else you would like to talk about, or anything that I have forgotten to ask about?

Thank you for your time. We will leave you with a list of organisations that you can contact if you need support.
